# Supplementary material for: Ceritinib-Induced Regression of an Insulin-Like Growth Factor-Driven Neuroepithelial Brain Tumor
Source: Int J Mol Sci. 2019 Aug 30;20(17):4267. doi: 10.3390/ijms20174267 (PMC6747232; doi:10.3390/ijms20174267)
Supplement: Supplementary file 1 [file ijms-20-04267-s001.zip › ijms-555808-proofed-supplementary/Supplementary data_rev.pdf]

Table S1. Transcripts of the Notch pathway deregulated in the first relapse.

Symbol	Entrez Gene Name	TPM normal	TPM tumor	Exp Fold Change	Expected	Location	Type(s)
CNTN1	contactin 1	199	1	-199	Up	Plasma Membrane	enzyme
DLL1	delta like canonical Notch ligand 1	5	238	48	Up	Plasma Membrane	enzyme
DLL3	delta like canonical Notch ligand 3	5	105	21	Up	Extracellular Space	other
DTX4	deltex E3 ubiquitin ligase 4	31	2	-15		Cytoplasm	enzyme
HES1	hes family bHLH transcription factor 1	11	160	14		Nucleus	transcription regulator
HES7	hes family bHLH transcription factor 7	1	58	58		Nucleus	transcription regulator
JAG1	jagged 1	9	95	11	Up	Extracellular Space	growth factor
LFNG	LFNG O-fucosylpeptide 3-beta-N-acetylglucosaminyltransferase	10	741	74	Down	Cytoplasm	enzyme
MAG	myelin associated glycoprotein			-440.212		Plasma Membrane	other
NOTCH1	notch 1	7	183	26	Up	Plasma Membrane	transcription regulator
NUMBL	NUMB like, endocytic adaptor protein	50	4	-12	Down	Cytoplasm	other

The Exp fold change indicates the ratio between the TPMs of the first relapse (TPM tumor) and of the normal brain (TPM normal, sample 111). If known, the expected status (up regulated or down regulated) if the pathway is activate is indicated. The location and function (Type) of the corresponding proteins are also indicated.

Table S2. Transcripts of the BBC pathway deregulated in the first relapse. See supplementary Table 1 for legend.

Symbol	Entrez Gene Name	TPM normal	TPM tumor	Exp Fold Change	Expected	Location	Type(s)
APC2	APC2, WNT signaling pathway regulator	33	1	-33		Cytoplasm	enzyme
BMP4	bone morphogenetic protein 4	2	26	13	Up	Extracellular Space	growth factor
BMP8B	bone morphogenetic protein 8b	2	96	48	Up	Extracellular Space	growth factor
FZD10	frizzled class receptor 10	1	31	31	Up	Plasma Membrane	G-protein coupled receptor
FZD2	frizzled class receptor 2	1	23	23	Up	Plasma Membrane	G-protein coupled receptor
GLI2	GLI family zinc finger 2	1	25	25	Up	Nucleus	transcription regulator
GLIS1	GLIS family zinc finger 1	1	30	30	Up	Nucleus	transcription regulator
HHIP	hedgehog interacting protein	13	1	-13	Up	Plasma Membrane	other
KIF7	kinesin family member 7	2	20	10	Up	Extracellular Space	other
PTCH2	patched 2	3	44	15		Plasma Membrane	transmembrane receptor
WNT10B	Wnt family member 10B	28	1	-28	Up	Extracellular Space	other
WNT3A	Wnt family member 3A	1	44	44	Up	Extracellular Space	cytokine
WNT5A	Wnt family member 5A	3	56	19	Up	Extracellular Space	cytokine
WNT7A	Wnt family member 7A	10	1	-10	Up	Extracellular Space	cytokine

Table S3. Expression of IGF2, IGF1R and INSR in first relapse compared to normal brain.

Symbol	Entrez Gene Name	TPM normal	TPM tumor	Exp Fold Change
IGF2	insulin like growth factor 2	6	1442	240
IGF1	insulin like growth factor 1	4	2	-2
IGF1R	insulin-like growth factor 1 receptor	11	3	-4
INSR	insulin like growth factor 2	13	34	3
