# Supplementary material for: Ceritinib-Induced Regression of an Insulin-Like Growth Factor-Driven Neuroepithelial Brain Tumor
Source: Int J Mol Sci. 2019 Aug 30;20(17):4267. doi: 10.3390/ijms20174267 (PMC6747232; doi:10.3390/ijms20174267)
Supplement: Supplementary file 1 [file ijms-20-04267-s001.zip › ijms-555808-proofed-supplementary/supplementary Figure 1.pdf]

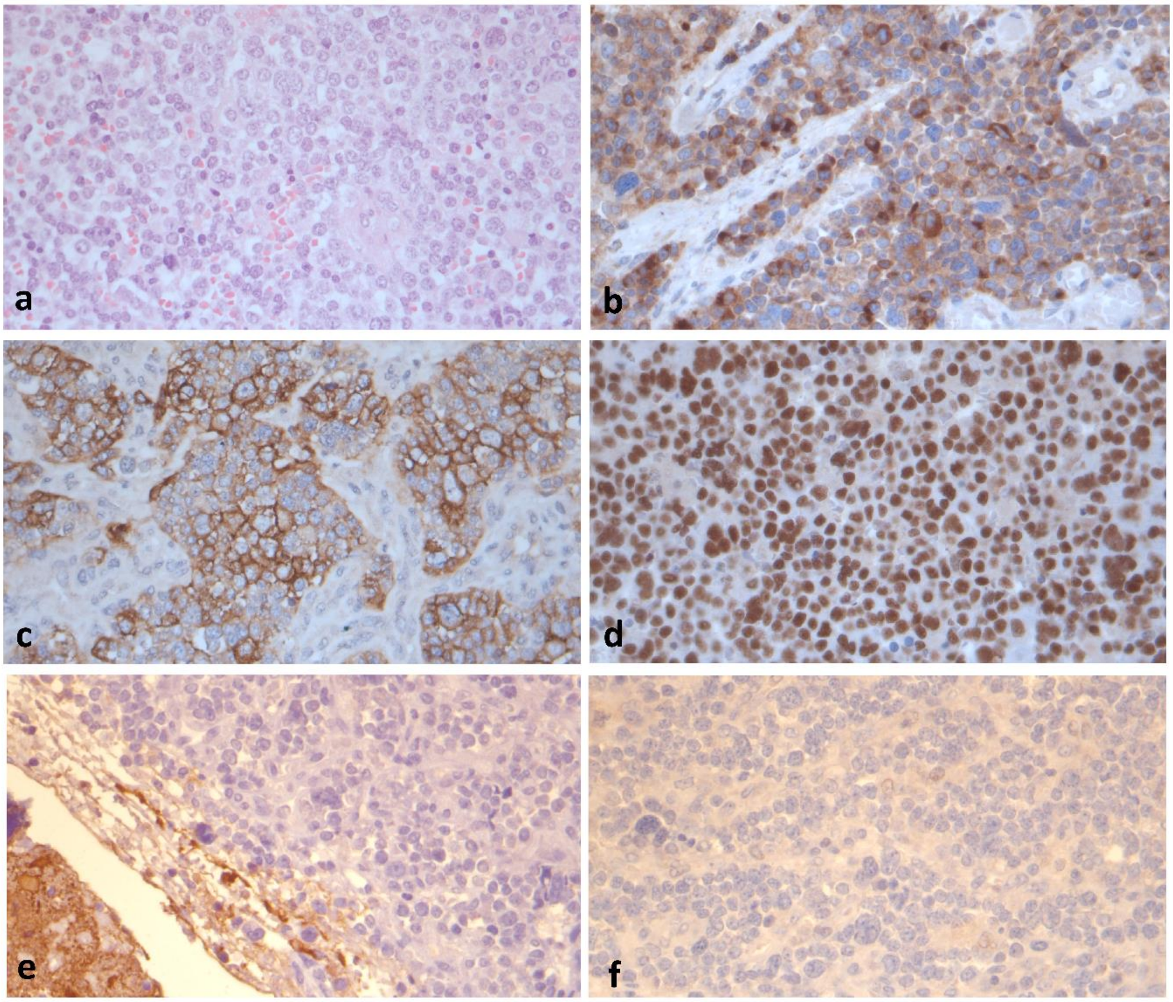

Figure S1. Histopathological features of the relapse. H&E staining shows an undifferentiated cellular tumor lacking characteristic growth patterns (a). Most tumor cells were positive for EMA, sometimes displaying a dot-like positivity (b). Tumor cells further showed L1CAM-positivity (c). Nuclear INI1 expression was retained (d). GFAP (e) and IDH1 R132H (f) were not expressed. Magnification 400X.
