# Supplementary material for: Ceritinib-Induced Regression of an Insulin-Like Growth Factor-Driven Neuroepithelial Brain Tumor
Source: Int J Mol Sci. 2019 Aug 30;20(17):4267. doi: 10.3390/ijms20174267 (PMC6747232; doi:10.3390/ijms20174267)
Supplement: Supplementary file 1 [file ijms-20-04267-s001.zip › ijms-555808-proofed-supplementary/Supplementary material and methods_rev.pdf]

Ethical standards

The study delineates the administration of a pharmaceutical drug for an unapproved indication in an unapproved age group (off-label use). The purpose of off-label use is to benefit the individual patient and therapeutic decision-making is guided by the best available evidence and the importance of the benefit for the individual patient. In our patient, that suffered from a life-threatening disease, the off-label use of Ceritinib was performed as an individual therapeutic approach comparable to a compassionate use (except that Ceritinib is a drug approved by the EMA e.g. for the therapy in ALK-rearranged NSCLC in adults) lacking any other remaining evidence based treatment options. The European Medicines Agency (EMA)'s Guideline on Compassionate Use of Medicinal Products, Pursuant to Article 83 of Regulation (EC) No 726/2004, developed by the Committee for Medicinal Products for Human Use (CHMP) [1], states that compassionate use is performed primarily for therapeutic purposes of "patients with a chronically or seriously debilitating disease, or a life threatening disease, and who cannot be treated satisfactorily by an authorized medicinal product" in the European Union, of an individual patient. Thus, by its very nature, compassionate use represents a kind of treatment and not biomedical research [2], especially if not performed as a compassionate use series or program. Off-label and compassionate use regulations have already been introduced in Germany and do not require independent ethical review. The main focus of the German research ethics committee's (REC) is to protect the rights, dignity and safety of biomedical research participants within clinical trials. Therefore, off-label and compassionate use concerning the treatment of an individual patient does not fall within the scope of interest of these committees.

In our case, the whole team and especially the physicians, who were responsible for the treatment, were regularly trained in Good Clinical Practice (GCP) standards and long-term experienced in conducting clinical trials. Furthermore, all treating physicians were qualified pediatric oncologists and members of the German pediatric society for hematology and oncology (GPOH) with raised awareness of the ethical aspects of compassionate use.

In order to protect the patient and to improve knowledge about medicine used in conditions other than those authorised, this patient was closely (weekly) observed, suspected adverse drug reactions (ADR) were reported in a timely fashion, and safety signals were identified and monitored.

The informed consent process comprised a multi-step-procedure concerning the enlightenment of the parents by the medical team in the presence of an innocent bystander and a non-medical-versed native speaker for the translation into english for the father. Following each session, the parents were proactively encouraged to obtain a second opinion. This approach was utilized twice.

We used informed consent forms that are standardized, especially designed for off-label use, approved for the application in our institution and commercially available (Thieme Compliance GmbH, Erlangen, Germany 2017 version 10/72017v1, order number DE613126).

Prior to the enlightening of the parents, the treatment was discussed with colleagues of the German relapse brain tumor study coordination office, HIT Rez, Essen, Germany. This statement, determining the favorable risk: benefit ratio, as well as the biological *in vitro* data, constituted the basis for an application for the cost takeover by the patients health care insurance.

German law includes some specific provisions on off-label use in the regulation on health insurance (Paragraph 275 social secure code, §275 SGB V [3]: therefore, a special medical advisory service of the German association of the statutory health insurance funds (MDK), consisting of independent experts in oncology, reviewed the application. Finally, their statement led to a positive vote for the cost

takeover by the patients health care insurance. So, the decision for using ceritinib was made independently of any commercial sales interests of the drug manufacturer.

Concerning the anonymity of the patient, samples were stored in Pseudonymization form. Pseudonymization adds an additional layer of protection for person-related data. It has been implemented as important security measure in many projects including the German National Cohort. Only authorized persons have access to the data.

Literatur

1 https://www.ema.europa.eu/en/documents/regulatory-procedural-guideline/guideline-compassionate-use-medicinal-products-pursuant-article-83-regulation-ec-no-726/2004_en.pdf Accessed 27.7.2019

2 Borysowski et al. BMC Medicine (2017) 15:136
DOI 10.1186/s12916-017-0910-9.

3 https://www.gesetze-im-internet.de/sgb_5/__275.html accessed 27.07.2019
