# Supplementary material for: Ceritinib-Induced Regression of an Insulin-Like Growth Factor-Driven Neuroepithelial Brain Tumor
Source: Int J Mol Sci. 2019 Aug 30;20(17):4267. doi: 10.3390/ijms20174267 (PMC6747232; doi:10.3390/ijms20174267)
Supplement: Supplementary file 1 [file ijms-20-04267-s001.zip › ijms-555808-proofed-supplementary/supplementary-Figure 2.pdf]

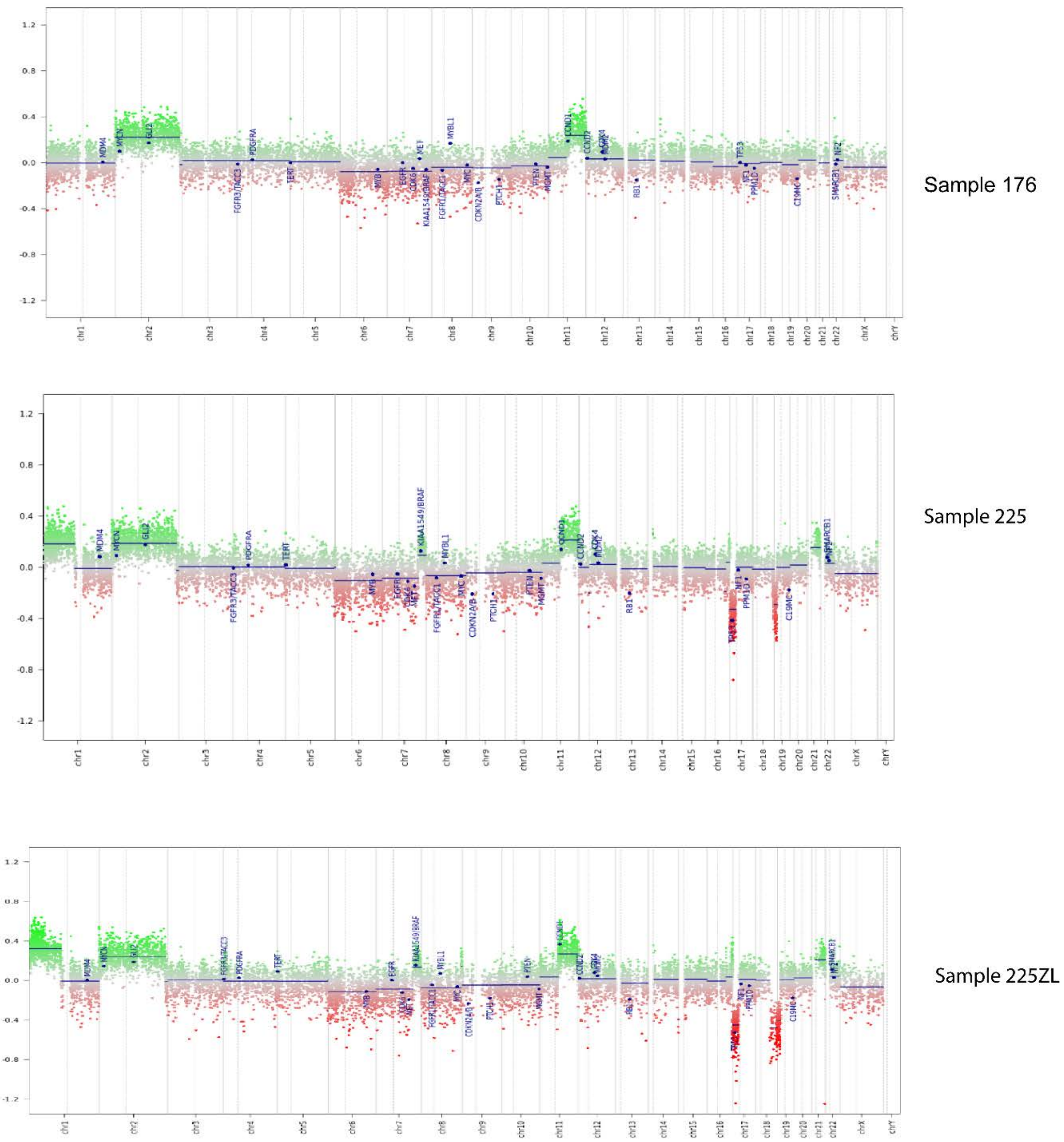

Figure S2. DNA copy-number alterations. Copy-number alterations of the primary tumor (176) the recurrence (225) and the primary tumor cells (225ZL) were analyzed using the EPIC 850k DNA methylome bead array.
